# Knowledge and Practice on Prevention and Control of Tuberculosis Among Nurses Working in a Regional Hospital, Nepal

**DOI:** 10.3389/fmed.2021.788833

**Published:** 2022-02-04

**Authors:** Mira Adhikari Baral, Sumitra Koirala

**Affiliations:** ^1^Adult Health Nursing Department, Institute of Medicine, Tribhuvan University, Pokhara, Nepal; ^2^Department of Orthopaedics, Manipal Teaching Hospital, Pokhara, Nepal

**Keywords:** personal protective equipment, ventilation, disinfection, TB transmission, nurses

## Abstract

**Introduction:**

Tuberculosis (TB) is a highly prevalent communicable disease in Nepal. All health personnel who care for such patients are at high risk of developing tuberculosis. It is very necessary for all healthcare providers, especially nurses, who spend a lot of time with such patients, to have adequate knowledge and correct practice on the occupational safety measures to maintain health and prevent transmission of the disease. This study was carried out to assess the knowledge and practice of nurses in the prevention and control of TB infection.

**Method:**

This study was a cross-sectional study conducted on nurses working in all wards and Outpatient Departments (OPDs) of Western Regional Hospital, Pokhara, Nepal. A complete enumeration of the respondents was performed, and data were collected consecutively, using a semi-structured self-administered questionnaire, from all nurses working in all the wards and OPDs of the hospital. The predictors of knowledge of TB infection prevention and control (TBIPC) were assessed using binary logistic regression.

**Results:**

The study findings showed that a majority of nurses had an inadequate level of knowledge and poor practice of prevention and control of TB. Regarding practice, none of the nurses reported the use of an N95 mask or a respirator during care of the patients with TB and all the nurses reported that they used chemical disinfectant (virex) to clean the room and the surfaces. However, none of them reported the use of fumigation or ultraviolet irradiation for disinfection. Nurses who were 40 years and older (*p* = 0.001, adjusted odds ratio (A*OR*) = 5.965, *CI* = 2.083–17.457) and who were currently working in the wards with isolation rooms demonstrated higher odds for knowledge on TBIPC (*p* = 0.010, A*OR* = 2.686, *CI* = 1.264–5.710).

**Conclusions:**

This study showed that a majority of nurses had an inadequate level of knowledge and implemented poor safety measures for the prevention and control of tuberculosis. This increases their risk of being infected with TB infection and cross-transmission. It is recommended that the hospital plan and conduct the necessary education/training for nurses to update their knowledge, develop the organizational structure and policies, and establish a system to support and monitor the practice of health workers on the prevention and control of TB, and maintain the health and safety of nurses caring for patients with TB.

## Introduction

Tuberculosis (TB) is a global health burden. TB is caused by *Mycobacterium tuberculosis* (MTB) that commonly affects the lungs (85%) and other body parts. It is the second leading infectious killer disease worldwide after COVID-19, leading above HIV/AIDS (1). It accounts for over 95% of the TB deaths in low- and middle-income countries and is highly prevalent in young people of the productive age group. TB is also the leading cause of death of people with HIV and a major contributor to the resistance to antimicrobials. A report by the WHO showed that a majority of the global number of TB cases are found in South East Asia with 211 cases per 1,00,000 population (1). In Nepal, TB is the seventh leading cause of death (2). TB, as a highly communicable disease, imposes a higher risk of cross-infection to healthcare workers (3–6), especially to the nurses who work on the front line (5–7), as well as to other patients during the hospital stay. The risk of TB cross-infection increases when personal, environmental, engineering, and administrative control measures are not well established (3, 8–10). Knowledge of TB infection prevention and control (TBIPC) measures among nurses and their implementation decrease the risk of cross-transmission. Various anecdotes show that in developing countries, engineering, environmental, administrative, and personal measures for the prevention and control of TB are not well maintained (11–13). In addition to this, the lack of regular screening of patients with HIV for TB and the lack of adequate isolation rooms in the hospital to isolate symptomatic patients with HIV for TB further increase the challenges for health workers to prevent the cross-infection between health workers and other patients (13). Moreover, this can result in hospital-acquired TB infection in admitted patients and health workers working in that arena. Studies show that health workers are at increased risk of hospitalization for multidrug-resistant and extensively drug-resistant TB compared with non-health workers (14, 15). TBIPC measures are the most cost-effective measures to reduce the TB burden. The knowledge of TBIPC measures among healthcare workers can enhance their practice of TBIPC. This can reduce and/or control the rates of cross-transmission of TB. However, various studies suggest that nurses possess limited knowledge and have a poor practice of TBIPC (16, 17).

Tuberculosis infection control is an essential component of the WHO Stop TB strategy for large reductions in TB incidence, TB deaths, and treatment costs faced by the patients with TB, and contribute to the strengthening of health systems. In Nepal, in line with the strategy, mass campaigns and treatments modules have been taken into action to reduce the burden of TB. The National Tuberculosis Center has made a significant effort for the treatment of patients with TB; however, monitoring of aspects of implementation, such as personal protection, environmental, engineering, and administrative safety measures in hospitals and primary care centers, is poorly considered (3). Very limited studies have been conducted to evaluate the knowledge and practice of TBIPC among health workers in Nepal, and these studies showed that health workers have very limited knowledge and poor practice of TBIPC of TB infections (18, 19). Specifically, no study has been conducted among nurses, who work in the front line with patients and care for patients with TB. The findings of this study could provide insight into the knowledge and practice of nurses on the prevention and control in a limited resource setting. This can provide a basis for the concerned authority to design appropriate interventions to fill the gaps to improve the knowledge and practices of the nurses on TBIPC.

## Materials and Methods

### Study Design, Setting, and Population

This was a facility-based cross-sectional study conducted among nurses working in the wards and OPDs of Western Regional Hospital. Western Regional Hospital is a government hospital of Nepal located in Ramghat, Pokhara, Nepal. It is a 350-beded hospital and is also a tertiary hospital for the western region of Nepal (16 districts). Based on available administrative data, 159 nurses were working in the hospital. All the nurses working in this hospital were women. This hospital provides services to patients with infectious diseases, such as tuberculosis and HIV/AIDS. This hospital has various wards that have separate isolation rooms. The isolation rooms have been engineered in such a way that their entrance (door) faces directly to another open unit in the ward.

#### Sampling Method and Sample Size

**Figure 1:**
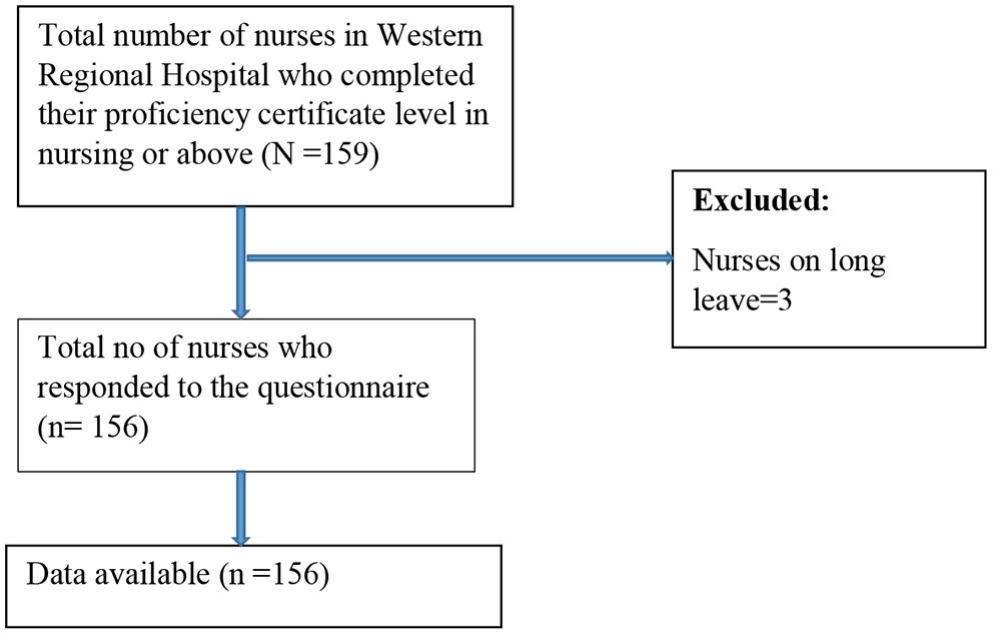
Selection of participants for the study.

The study population was nurses (including volunteer nurses) working in the wards and OPDs of Western Regional Hospital who had completed their proficiency certificate Level in nursing or higher from a recognized institution and had their nursing license. There were altogether 159 nurses working at Western Regional Hospital and all of them were women. A complete enumeration of the nurses was performed. However, three nurses were on long-term leave and thus were excluded from the study. Together, 156 nurses participated in the study; the response rate was 100% (Figure 1).

### Tools and Techniques for Data Collection

#### Data Collection Tool

The tool used for the study was a semi-structured self-administered questionnaire developed by the researchers themselves after an intense literature review. The tool was based on the guidelines for the prevention and control of TB infection in the healthcare setting developed by the WHO and adopted by the Nepal Ministry of Health (1, 2). The content validation of the tool was done by a panel of three infectious disease experts working in the Western Regional Hospital and Manipal Teaching Hospital. The experts checked the tool for its ability to reflect key variables related to knowledge and practice on TBIPC. Some modification in the tool was made as per the experts' feedback. The tool was pretested on 16 nurses who met the inclusion criteria in the Manipal Teaching Hospital to assess the clarity, consistency, and adequacy of the tool. The internal consistency of the tool was analyzed by calculating Cronbach's alpha and the value of Cronbach's alpha for knowledge was 0.75 and that for practice was 0.67, which is within an acceptable value (20). The questionnaire consisted of 3 parts with a total of 26 questions, such as multiple-choice questions and multiple response questions. Part I consists of 6 questions on background information (age, education, currently working ward, work experience, training on infection prevention and control, and intake of any immunosuppressive drugs at present). Part II consisted of 13 questions related to knowledge on modes of TB transmission, and various personal protective, administrative, and environmental control measures for prevention and control of TB transmission. These included the questions on various activities and procedures that increase the risk of TB transmission, selection of appropriate masks by healthcare professionals for the protection from cough generated TB aerosols and the fit test, management of suspected patients with TB in the waiting area and the ward, physical distancing, ventilation, disposal of sputum, and disinfection of isolation room. Part III consisted of seven questions related to the practice of TBIPC among nurses. Specifically, it included questions on mask use during the care of patients with TB and suspected patients with TB, the types of masks used, the frequency with the mask used, and the method of disinfection of isolation rooms.

#### Data Collection Procedure

The study was carried out after ethical clearance of the research proposal by the Institutional Review Committee of the Institute of Medicine at Tribhuvan University. Then, formal permission was obtained from the concerned hospital authority for data collection. Data were collected in July 2018. Before data collection, the participants were explained the purpose and nature of the study. They were informed that they had full authority to withdraw from the study without fear and explanation at any time during data collection. Then, informed written consent was obtained from the nurses and the questionnaire was distributed to them at their convenient time at their workplace and the completed questionnaire was collected the same day. The average time required to complete the questionnaire was 20 min. The filled questionnaire was kept confidential using code to link the respondent to the questionnaire and the records were kept confidential. The data obtained were used only for research purposes.

#### Data Processing and Analysis

Data were collected from 156 respondents. The collected data were edited at the data collection site to find errors and omissions and corrected by verifying or filling the data with the respondent regarding the questionnaire. The data were then organized, coded, and entered into the Statistical Package for Social Sciences (SPSS) version 16.

The correct answer on each of the multiple-choice questions (MCQ) received the weightage of score 1, while for the multiple-response types of question (MRQ), the weightage score of 1 was allocated to each alternative, and the correct response to each alternative was given the score of 1. Since the data were found to be normally distributed, the mean was used to classify the level of knowledge. The level of knowledge was determined by calculating the mean score of the total correct response/s on each question (MCQ/MRQ) related to the knowledge, and the mean score of the knowledge of the nurses was calculated which was 13.39. The score below the mean was considered as the inadequate level of knowledge, while the mean and above the mean were considered as the adequate level of knowledge (21). Regarding the practice, the correct response to the questions related to practice was given a weightage score of 1. The level of significance was considered at a *p* < 0.05. Among the practice questions, three questions were only limited to those nurses who cared for a patient with pulmonary TB in the hospital.

Data were then analyzed using descriptive statistics, such as frequency, percentage, mean, and SD. Binary logistic regression was used as inferential statistics to identify the factors associated with the knowledge of nurses on the prevention and control of TB infections.

#### Limitation of the Study

This study was conducted in a single setting. Therefore, the findings cannot be generalized in all settings and populations. The responses to the knowledge questionnaire are based on the recall of learned facts and information, so there is a chance of recall bias. Furthermore, responses to the practice are based on the reports of nurses rather than direct observation. Thus, the finding of practice has limited generalizability.

## Results

This study revealed that most of the nurses were from the age group 19–39 years old (84.6%), and worked in the wards without isolation rooms (60.9%). Out of total nurses, 60.2% had completed Proficiency Certificate level nursing, 37.2% of the nurses had work experience of 1–5 years, 26.9% of the nurses had received training in infection prevention and control, and 8.3% of the nurses were taking immunosuppressive agents during data collection time (Table 1).

**Table 1:** Background characteristics of respondents *n* = 156.

**Background information**	**Frequency**	**Percentage**
**Age classification**		
19–39 years	132	84.6
40–60 years	24	15.4
**Currently working ward/OPD**		
With isolation rooms	61	39.1
Without isolation rooms	95	60.9
**Education**		
PCL Nursing	94	60.2
Bachelor of Nursing	55	35.3
Master of Nursing	7	4.5
**Work experience**		
<1 year	35	22.4
1–5 years	58	37.2
6–10 years	26	16.7
>10 years	37	23.7
**Training on infection prevention**		
Got training	42	26.9
No training	114	73.1
**Taking any immune-suppressive drugs at present**		
Yes	13	8.3
No	143	91.7

Regarding knowledge, all the nurses had the correct knowledge on coughing as a mode of spread of TB bacilli (100%). Concerning masks, 20.5% of nurses reported that the N95 mask offers protection against TB droplet nuclei, while 14.7% reported that FFP2 protects against TB droplet nuclei and 82.1% of the nurses reported that a surgical mask is protective against TB droplet nuclei. About the fit test, only 21.8% of the respondents reported that after wearing a mask, it should be checked if it forms a tight seal. Similarly, 41% of nurses were aware that the best method of ventilation in the isolation room is negative pressure ventilation. The entrance door of the isolation rooms in all the wards of the Western Regional Hospital face directly to another open unit in the ward. Provided the condition of the engineered isolation rooms in the Western Regional Hospital, only 5.8% of the respondents correctly answered the correct method to maintain ventilation in the room. About half of the nurses had correct knowledge on the disposal of sputum (50.6%), while only 28.8% of the respondents demonstrated correct knowledge on the correct disinfection methods of the isolation room after discharge of the patient with active pulmonary TB (Table 2). Overall, about one-third of nurses (34.6%) had adequate knowledge of TB prevention and control (Table 3).

**Table 2:** Aspects of knowledge of the prevention and control of tuberculosis (TB) among nurses *n* = 156.

**Aspects of knowledge**	**No. of correct responses**	**Percentage**
**Mask/masks that offer protection of health care workers against TB droplet nuclei**		
N95 mask	32	20.5
FFP_2_ respirator	23	14.7
Surgical mask (incorrect option)	28	17.9
Cloth mask (incorrect option)	31	19.9
**After wearing a mask, it should be tested if it forms a tight seal**	34	21.8
**Ventilation**		
The best Ventilation of isolation room is negative pressure ventilation	64	41
In the case when the isolation room has an entrance **door** that is **directly facing** the other open unit, and has a window facing an open area and has a ceiling fan, the correct method of ventilating the room is: closing the door and opening the windows, and turning on the fan to maintain ventilation.	9	5.8
Patients suspected of tuberculosis should be kept in well ventilated waiting area	104	66.7
**Personal and professional protection and administrative control**		
Ask patients suspected of tuberculosis to cough into tissue/handkerchief	53	34
Rotate staff working in the infectious unit to the noninfectious unit very frequently (incorrect option)	82	52.6
Consume a healthy diet	132	84.6
Ask the patient suspected of TB to wear a surgical mask	94	60.3
Wash hands before and after caring for the patient	126	80.8
**Distancing and Position**		
The least distance that should be maintained from the patient suspected of tuberculosis is 1 meter	93	59.6
The position of the health professional with the patient with tuberculosis must be L shaped during history and examination of the patient	127	81.4
**Modes of transmission of TB infection**		
Touching bed linens of patients increases the risk of TB transmission (incorrect option)	64	41.0
Eating on the same plate with the TB patient increases the risk of TB transmission (incorrect option)	84	53.8
Coughing by TB patient increases the risk of TB transmission	156	100
Sneezing by TB patient increases the risk of TB transmission	136	87.2
Kissing a TB patient increases the risk of TB transmission (incorrect option)	68	43.6
**Usually, the patient on TB regime is infectious until the first 2 weeks of initiating anti-tubercular treatment**	115	73.7
**Medical/surgical/diagnostic procedures that increase risk of cross-transmission of pulmonary tuberculosis to health care professionals**		
Suctioning	100	64.1
Intubation	74	47.4
Bronchoscopy	68	43.6
Dressing (incorrect option)	69	44.2
**Disposal of sputum and disinfection of the room**		
Sputum should be discarded into the tissue, then into the container and the container should be covered with a lid	79	50.6
After discharge of the active TB patient, disinfection of the room should be either done with Ultraviolet (UV) irradiation/fumigation.	45	28.8

**Table 3:** Level of knowledge of nurses about the prevention and control of TB *n* = 156.

**Knowledge**	**Frequency**	**Percentage**
Inadequate knowledge	102	65.4
Adequate Knowledge	54	34.6

*Mean knowledge = 13.39 ± 3.055 SD (Min score: 8, Max score: 22)*.

Similarly, all the nurses reported that they used a mask while providing care to the patient suspected of TB and the commonly used mask was a cloth mask. However, only 15.4% of the nurses reported using a surgical mask while caring for a patient suspected of TB, while none of them reported wearing an N95 or an FFP2 mask, and only 12.2% of the nurses reported that they always wore the mask while caring for a patient suspected of tuberculosis. Only 86.5% of nurses reported that they had cared for patients with pulmonary TB in the hospital and out of them, none reported using an N95 or FFP_2_ mask while caring for the patient, and all reported that they disinfected the room with chemical liquid/liquefied disinfectant (virex) (Table 4).

**Table 4:** Practice of TB infection and prevention among nurses *n* = 156.

**Practice**	**Frequency**	**Percentage**
**Wear a mask during care suspected of TB**	**156**	**100**
**Type of mask worn**		
Cloth only	92	59
Surgical only	24	15.4
Cloth/surgical	40	25.6
**Frequency of mask worn**		
Always	19	12.2
Often	66	42.3
Sometimes	65	41.7
Rarely	6	3.8
**Ever cared for a patient with pulmonary tuberculosis in the hospital**	**135**	**86.5**
**Use of mask while caring for patients with pulmonary tuberculosis in the hospital (*****n*** **= 135)**		
Cloth	7	5.2
Surgical	83	61.5
Cloth/surgical	45	33.3
**Method of disinfection of the room after the patient is discharged** **(***n*** = 135)**		
Disinfection of the room with liquid/liquefied chemical disinfectants (virex)	135	100

This study revealed that nurses who were 40 years or older (*p* = 0.001, adjusted odds ratio (A*OR*) = 5.965, *CI* = 2.083–17.457) demonstrated higher odds of knowledge than those <40 years. Furthermore, the nurses who were currently working in isolation units demonstrated greater knowledge than those who were not currently working in isolation units (*p* = 0.010, A*OR* = 2.686, *CI* = 1.264–5.710) (Table 5).

**Table 5:** Association of background information with the knowledge of nurses about prevention and control of TB *n* = 156.

**Characteristics**	***p*** **-value**	**AOR#**	**CI**
**Age**			
19–39 years (Ref)			
40 years and above	0.001*	5.965	2.083–17.457
**Level of education**			
PCL Nursing (Ref)			
Bachelor and above	0.129	1.797	0.843–3.831
**Years of work experience**			
Up to 5 years (Ref)			
More than 5 years	0.324	1.518	0.662–3.481
**Working wards/units at present**			
Wards/units without isolation room (Ref)			
Wards/Units with isolation rooms	0.010*	2.686	1.264–5.710
**Training on infection prevention and control**			
Not received training (Ref)			
Received training	0.302	1.565	0.669–3.661

* *p < 0.05*.

# *The variable included in the adjustment model were age, level of education, years of work experience, working ward, and training on infection prevention and control*.

## Discussion

Tuberculosis is one of the common infectious diseases prevalent in Nepal. In Nepal, at the TB treatment sites, the bed occupancy rate of TB patients is 83% and the average length of stay is 11 days (21). Healthcare workers, who care for those patients, are at high risk of TB infection. However, the infection can be controlled to a greater extent if healthcare workers have adequate knowledge on TB prevention and control and implement their knowledge into practice.

This study shows that a very low proportion of nurses (34.6%) had adequate knowledge on the prevention and control of TB. In addition, they had poor knowledge of standard masks against TB aerosol, with 82.1% reporting that a wearing surgical mask can protect them from cough-generated TB aerosol. Only 21.8% of the nurses had correct knowledge on fittest, 5.8% had correct knowledge on ventilating the isolation rooms, and 28.8% had correct knowledge on disinfecting the isolation rooms (28.8%). Inadequate knowledge can be a key factor in affecting the practice of nurses on TBIPC, and therefore, they are at increased risk of acquiring and/or transmitting TB infection. This finding is consistent with other studies (22–24). This may be attributed to the lack of regular in-service education and the improper placement of nurses according to the training received and their specialty. Furthermore, the absence of the hospital's TBIPC policy could have decreased the focus of the administration to train nurses and implement other additional actions to increase the knowledge of nurses about the prevention and control of TB. Therefore, it is necessary to provide regular training to nurses in TBIPC and make judicious placement of nurses in the wards according to the specialty and training they received. It is recommended that the hospital implements the TBIPC policy to initiate and/or enforce various administrative, engineering, environmental, and personal protective measures to ensure the safety of health professionals during the care of patients with TB. Furthermore, the gap in nursing education and practice in Nepal (25) could have resulted in the poor reinforcement of the learned knowledge, resulting in inadequate knowledge. However, the findings contradict with studies conducted among nurses and/or various health workers in Lesotho, Uganda, and Malaysia, which showed that most of the health workers had adequate knowledge of prevention and control of TB infection (26–28). The inconsistency in the result can be attributed to differences in the characteristics of the sample and the setting. In addition, differences in the lower cut-off points used to classify good/adequate knowledge in these studies (26–28) may have resulted in better knowledge in those studies. This study shows that all nurses wore a mask while caring for the patient suspected of TB while none of the nurses wore an N95 or an FFP2 mask during the care of the patient; they wore a surgical or a cloth mask. Of the 135 nurses who reported caring for patients with pulmonary TB in that hospital, the majority (61.5%) reported using the surgical mask while caring for the patient with pulmonary TB, while none of them reported wearing an N95 mask while caring for such patients. The findings contradict other studies (19, 23, 29–31). The inconsistency of the result may be attributed to the differences in setting. Western Regional Hospital is a general government hospital and there is no separate infectious ward in the hospital. Furthermore, government hospitals of Nepal must work with a small budget and limited resources in the abundance of communicable diseases. The N95 mask cost relatively much more than a surgical mask, and the lack of hospital TBIPC policy may have resulted in less attention from the management team to maintain an adequate supply of personal protective equipment. This may have led to a poor/no supply of N95 masks to hospital nurses. Regarding room disinfection, 135 nurses out of 156 nurses who had reported having experience of caring for a patient with pulmonary TB, reported that they used chemical liquid/liquefied solution (virex) to disinfect the room. This kind of room disinfection does not kill aerosols and increases the risk of TB cross-transmission. Thus, health workers as well as the other patients admitted to that isolation room are at risk of cross-transmission. It is recommended that during the development of the TBIPC policy, the hospital includes regulations and procedures regarding disinfection of the isolation room in that policy.

This study shows that nurses who were 40 years old and above demonstrated higher odds of knowledge than those under 40 years of age (*p* = 0.001, A*OR* = 5.965, *CI* = 2.083–17.457). This finding is consistent with other studies which showed that increasing age is related to good knowledge (29, 32). Nurses aged 40 years or over are usually nurses working in the hospital who are at the senior levels of the ward and those who have greater priority and access to various facilities, such as in-service education and training. Furthermore, senior nurses have greater chances of being exposed to various isolation units of wards during their job tenure, where they would have exercised some practices for TBIPC that could have increased their knowledge about prevention and control of TB. This study showed that nurses who were currently working in isolation units demonstrated higher knowledge odds than those who were not currently working in isolation units (*p* = 0.010, A*OR* = 2.86, *CI* = 1.264–5.710). This may be related to the strengthened knowledge based on the practices, the nurse carries out to prevent cross-transmission TB. On the contrary, the level of education and training were not predictors of knowledge in this study. This finding is consistent with a study conducted in South Africa that showed that there is no relationship between TB infection prevention knowledge and training (23), while the finding contradicts the study in Ethiopia that showed that good knowledge was associated with TB infection prevention and control (TB-IPC) training (29). This may be related to the differences in the sample and setting. Furthermore, the mismatch of the assigned role of a nurse with her education and training in Nepal that institutes poor implementation of the learned knowledge and, therefore, poor recall of the learned knowledge, and the gap in nursing education and practice in Nepal (25) may have resulted in the poor knowledge of nurses about the prevention and control of TB infections. This calls for an improvement in the knowledge of nurses through frequent in-service education and reform in the education system; minimizing the gap in learned knowledge and practice, focusing on the practical application of the learned knowledge and skill development, and placing the nurses on their jobs according to their education, training, and specialty.

## Conclusion

This study showed that the level of knowledge and practice of nurses in the prevention and control of TB infection was inadequate and the level of knowledge was higher among the nurses who were 40 years and above, and who were currently working in the isolation units. It is recommended that the hospital plan and conduct the necessary education/training of nurses on the prevention and control of TB to update their knowledge. Additionally, it is necessary to develop and implement the TBIPC policy in the hospital and implement the placement provisions of nurses according to their training and specialty. It is recommended that the National Tuberculosis Center develops a mechanism to facilitate administrative, engineering, environmental, and personal protective measures for the prevention and control of TB in institutions that provide TB services and develops a mechanism for the supervision and monitoring of the measures.

## Data Availability Statement

The raw data supporting the conclusions of this article will be made available by the authors, without undue reservation.

## Ethics Statement

The studies involving human participants were reviewed and approved by Institutional Review Committee, Tribhuvan University, Institute of Medicine. The patients/participants provided their written informed consent to participate in this study.

## Author Contributions

MB: proposal development, tool development, data collection, data analysis, and writing report. SK: proposal development and writing report.

## Conflict of Interest

The authors declare that the research was conducted in the absence of any commercial or financial relationships that could be construed as a potential conflict of interest.

## Publisher's Note

All claims expressed in this article are solely those of the authors and do not necessarily represent those of their affiliated organizations, or those of the publisher, the editors and the reviewers. Any product that may be evaluated in this article, or claim that may be made by its manufacturer, is not guaranteed or endorsed by the publisher.
