# Supplementary material for: Knowledge and Practice on Prevention and Control of Tuberculosis Among Nurses Working in a Regional Hospital, Nepal
Source: Front Med (Lausanne). 2022 Feb 4;8:788833. doi: 10.3389/fmed.2021.788833 (PMC8864556; doi:10.3389/fmed.2021.788833)
Supplement: Supplementary file 1 [file Data_Sheet_1.PDF]

		surgical m	cloth masl	knowledg	Knowledg	knowledg	ventillatic	door of ro	suspected	respirator	rotate wa	Healthy di	Have pt su	distance f	handwash	position L	patient wi	touching t	eating san	coughing i	sneezing a	kissing no	oral care a	bronchosc	intubation	dressing n	knowledg	how to dis
surgical m	Pearson C	1	-0.107	.672**	.512**	.239**	-0.051	0.099	0.047	-0.018	0.143	-0.032	0.039	0.147	0.101	0.052	-0.024	0.051	0.098	.a	-0.07	0.027	0.106	.162*	0.124	0.021	-0.039	-.224**
	Sig. (2-tailed)		0.182	0	0	0.003	0.531	0.218	0.558	0.823	0.074	0.691	0.633	0.068	0.209	0.521	0.763	0.524	0.224	.	0.382	0.74	0.187	0.044	0.122	0.798	0.625	0.005
	N	156	156	156	156	156	156	156	156	156	156	156	156	156	156	156	156	156	156	156	156	156	156	156	156	156	156	156
cloth mas	Pearson C	-0.107	1	-0.054	-0.116	0.009	0.107	-0.123	0.045	0.084	-0.074	-0.099	0.076	-.212**	-0.002	-.175*	.188*	-0.154	-.216**	.a	0.095	-0.049	0.071	0.081	0.009	0.009	-0.087	-0.033
	Sig. (2-tail	0.182		0.503	0.148	0.907	0.183	0.125	0.573	0.299	0.36	0.217	0.345	0.008	0.985	0.029	0.019	0.055	0.007	.	0.239	0.543	0.377	0.317	0.906	0.908	0.282	0.679
	N	156	156	156	156	156	156	156	156	156	156	156	156	156	156	156	156	156	156	156	156	156	156	156	156	156	156	156
knowledg	Pearson C	.672**	-0.054	1	.460**	0.155	-0.036	0.079	0.056	-0.029	0.037	-0.047	0.121	0.03	0.087	-0.084	-0.021	-0.036	0.056	.a	-0.043	0.066	0.049	.194*	0.058	0.027	-0.038	-0.113
	Sig. (2-tail	0	0.503		0	0.054	0.652	0.33	0.486	0.717	0.642	0.557	0.134	0.711	0.282	0.299	0.792	0.652	0.485	.	0.597	0.415	0.542	0.015	0.473	0.738	0.635	0.159
	N	156	156	156	156	156	156	156	156	156	156	156	156	156	156	156	156	156	156	156	156	156	156	156	156	156	156	156
Knowledg	Pearson C	.512**	-0.116	.460**	1	.218**	-.163*	0.13	-0.013	-0.107	-0.039	-0.073	0.079	0.084	0.019	-0.034	-0.08	-0.053	0.022	.a	-0.111	0.072	0.047	.254**	.184*	-0.006	0.013	0.015
	Sig. (2-tail	0	0.148	0		0.006	0.042	0.106	0.874	0.182	0.625	0.364	0.326	0.295	0.81	0.677	0.319	0.513	0.782	.	0.168	0.372	0.557	0.001	0.021	0.938	0.874	0.857
	N	156	156	156	156	156	156	156	156	156	156	156	156	156	156	156	156	156	156	156	156	156	156	156	156	156	156	156
knowledg	Pearson C	.239**	0.009	0.155	.218**	1	0.033	0.069	0.077	-0.084	0.035	-0.033	0.111	-0.009	-0.058	-0.027	0.139	0.065	-0.041	.a	-0.03	0.037	.168*	0.131	0.058	0.061	0.024	-0.096
	Sig. (2-tail	0.003	0.907	0.054	0.006		0.681	0.391	0.34	0.299	0.664	0.682	0.166	0.916	0.475	0.737	0.084	0.422	0.614	.	0.712	0.647	0.036	0.103	0.47	0.447	0.763	0.232
	N	156	156	156	156	156	156	156	156	156	156	156	156	156	156	156	156	156	156	156	156	156	156	156	156	156	156	156
ventillatic	Pearson C	-0.051	0.107	-0.036	-.163*	0.033	1	-0.039	-.212**	0.117	-0.017	-0.078	-0.122	0.102	0.043	0.097	0.084	-0.033	-0.038	.a	0.047	0.029	-0.055	-0.05	-0.062	0.018	0.094	0.073
	Sig. (2-tail	0.531	0.183	0.652	0.042	0.681		0.632	0.008	0.145	0.836	0.334	0.131	0.204	0.592	0.228	0.3	0.68	0.636	.	0.56	0.72	0.495	0.536	0.445	0.822	0.245	0.365
	N	156	156	156	156	156	156	156	156	156	156	156	156	156	156	156	156	156	156	156	156	156	156	156	156	156	156	156
door of ro	Pearson C	0.099	-0.123	0.079	0.13	0.069	-0.039	1	0.058	.287**	-0.04	-0.123	-0.08	-0.02	-.158*	-0.023	0.023	-0.039	0.008	.a	-0.152	0.06	0.071	0.06	-0.015	0.001	0.134	-0.036
	Sig. (2-tail	0.218	0.125	0.33	0.106	0.391	0.632		0.47	0	0.618	0.126	0.321	0.8	0.048	0.775	0.777	0.632	0.916	.	0.058	0.459	0.382	0.459	0.854	0.989	0.095	0.654
	N	156	156	156	156	156	156	156	156	156	156	156	156	156	156	156	156	156	156	156	156	156	156	156	156	156	156	156
suspected	Pearson C	0.047	0.045	0.056	-0.013	0.077	-.212**	0.058	1	-0.038	0.036	0.151	.204*	0.055	0.138	0.117	.227**	0.009	0.055	.a	0.014	-0.009	.180*	0.018	0.073	0.055	-0.1	-.210**
	Sig. (2-tail	0.558	0.573	0.486	0.874	0.34	0.008	0.47		0.635	0.653	0.06	0.011	0.492	0.086	0.147	0.004	0.909	0.499	.	0.867	0.91	0.025	0.821	0.368	0.497	0.215	0.008
	N	156	156	156	156	156	156	156	156	156	156	156	156	156	156	156	156	156	156	156	156	156	156	156	156	156	156	156
respirator	Pearson C	-0.018	0.084	-0.029	-0.107	-0.084	0.117	.287**	-0.038	1	.248**	0.043	0.002	0.039	0.041	0.064	0.029	.172*	0.04	.a	0.073	-0.057	0.001	0.024	0.077	0.015	-0.023	0.021
	Sig. (2-tail	0.823	0.299	0.717	0.182	0.299	0.145	0	0.635		0.002	0.592	0.982	0.631	0.612	0.424	0.723	0.032	0.623	.	0.367	0.477	0.993	0.761	0.336	0.851	0.778	0.792
	N	156	156	156	156	156	156	156	156	156	156	156	156	156	156	156	156	156	156	156	156	156	156	156	156	156	156	156
rotate wa	Pearson C	0.143	-0.074	0.037	-0.039	0.035	-0.017	-0.04	0.036	.248**	1	0.093	-0.063	.238**	0.155	.173*	0.045	.401**	0.073	.a	0.058	0.084	0.119	0.084	0.105	-0.033	-0.116	-0.019
	Sig. (2-tail	0.074	0.36	0.642	0.625	0.664	0.836	0.618	0.653	0.002		0.248	0.433	0.003	0.053	0.031	0.575	0	0.363	.	0.471	0.295	0.14	0.295	0.19	0.684	0.149	0.818
	N	156	156	156	156	156	156	156	156	156	156	156	156	156	156	156	156	156	156	156	156	156	156	156	156	156	156	156
Healthy d	Pearson C	-0.032	-0.099	-0.047	-0.073	-0.033	-0.078	-0.123	0.151	0.043	0.093	1	0.126	0.12	0.062	.207**	0.068	0.067	-0.038	.a	0.102	0.052	-0.023	-0.055	0.014	-0.014	0.005	-0.003
	Sig. (2-tail	0.691	0.217	0.557	0.364	0.682	0.334	0.126	0.06	0.592	0.248		0.118	0.136	0.439	0.009	0.397	0.408	0.634	.	0.204	0.516	0.778	0.494	0.865	0.865	0.946	0.97
	N	156	156	156	156	156	156	156	156	156	156	156	156	156	156	156	156	156	156	156	156	156	156	156	156	156	156	156
Have pt su	Pearson C	0.039	0.076	0.121	0.079	0.111	-0.122	-0.08	.204*	0.002	-0.063	0.126	1	-0.001	0.102	0.05	.170*	0.038	-0.016	.a	0.041	-0.079	-0.034	0.133	0.037	-0.015	-0.121	-0.061
	Sig. (2-tail	0.633	0.345	0.134	0.326	0.166	0.131	0.321	0.011	0.982	0.433	0.118		0.99	0.204	0.538	0.034	0.636	0.841	.	0.61	0.33	0.671	0.099	0.647	0.85	0.134	0.448
	N	156	156	156	156	156	156	156	156	156	156	156	156	156	156	156	156	156	156	156	156	156	156	156	156	156	156	156
distance f	Pearson C	0.147	-.212**	0.03	0.084	-0.009	0.102	-0.02	0.055	0.039	.238**	0.12	-0.001	1	0.062	.346**	.162*	.288**	-0.002	.a	0.153	0.144	0.119	0.144	0.075	-0.004	0.102	-.168*
	Sig. (2-tail	0.068	0.008	0.711	0.295	0.916	0.204	0.8	0.492	0.631	0.003	0.136	0.99		0.438	0	0.044	0	0.98	.	0.056	0.073	0.138	0.073	0.349	0.965	0.205	0.036
	N	156	156	156	156	156	156	156	156	156	156	156	156	156	156	156	156	156	156	156	156	156	156	156	156	156	156	156
handwash	Pearson C	0.101	-0.002	0.087	0.019	-0.058	0.043	-.158*	0.138	0.041	0.155	0.062	0.102	0.062	1	.269**	0.152	0.076	0.136	.a	0.007	-.161*	0.076	0.035	0.138	-0.057	-0.026	-0.12
	Sig. (2-tail	0.209	0.985	0.282	0.81	0.475	0.592	0.048	0.086	0.612	0.053	0.439	0.204	0.438		0.001	0.058	0.344	0.092	.	0.926	0.044	0.348	0.662	0.086	0.482	0.745	0.135
	N	156	156	156	156	156	156	156	156	156	156	156	156	156	156	156	156	156	156	156	156	156	156	156	156	156	156	156
position L	Pearson C	0.052	-.175*	-0.084	-0.034	-0.027	0.097	-0.023	0.117	0.064	.173*	.207**	0.05	.346**	.269**	1	.164*	.198*	0.12	.a	.162*	0.088	.192*	0.021	-0.008	-0.006	0.089	-.205*
	Sig. (2-tail	0.521	0.029	0.299	0.677	0.737	0.228	0.775	0.147	0.424	0.031	0.009	0.538	0	0.001		0.041	0.013	0.137	.	0.044	0.276	0.016	0.792	0.921	0.943	0.272	0.01
	N	156	156	156	156	156	156	156	156	156	156	156	156	156	156	156	156	156	156	156	156	156	156	156	156	156	156	156
patient w	Pearson C	-0.024	.188*	-0.021	-0.08	0.139	0.084	0.023	.227**	0.029	0.045	0.068	.170*	.162*	0.152	.164*	1	0.024	0.002	.a	.337**	-0.063	0.13	.172*	-0.016	-0.113	0.08	-.231**
	Sig. (2-tail	0.763	0.019	0.792	0.319	0.084	0.3	0.777	0.004	0.723	0.575	0.397	0.034	0.044	0.058	0.041		0.763	0.978	.	0	0.438	0.106	0.031	0.842	0.159	0.318	0.004
	N	156	156	156	156	156	156	156	156	156	156	156	156	156	156	156	156	156	156	156	156	156	156	156	156	156	156	156
touching t	Pearson C	0.051	-0.154	-0.036	-0.053	0.065	-0.033	-0.039	0.009	.172*	.401**	0.067	0.038	.288**	0.076	.198*	0.024	1	0.145	.a	0.047	0.055	0.135	0.082	.173*	0.097	0.015	-0.1
	Sig. (2-tail	0.524	0.055	0.652	0.513	0.422	0.68	0.632	0.909	0.032	0	0.408	0.636	0	0.344	0.013	0.763		0.071	.	0.56	0.493	0.093	0.312	0.03	0.229	0.849	0.216
	N	156	156	156	156	156	156	156	156	156	156	156	156	156	156	156	156	156	156	156	156	156	156	156	156	156	156	156
eating san	Pearson C	0.098	-.216**	0.056	0.022	-0.041	-0.038	0.008	0.055	0.04	0.073	-0.038	-0.016	-0.002	0.136	0.12	0.002	0.145	1	.a	-0.086	-0.094	0.085	-0.016	-0.048	0.151	-0.117	-0.063
	Sig. (2-tail	0.224	0.007	0.485	0.782	0.614	0.636	0.916	0.499	0.623	0.363	0.634	0.841	0.98	0.092	0.137	0.978	0.071		.	0.287	0.244	0.294	0.843	0.556	0.059	0.147	0.432
	N	156	156	156	156	156	156	156	156	156	156	156	156	156	156	156	156	156	156	156	156	156	156	156	156	156	156	156
coughing	Pearson C	.a	.a	.a	.a	.a	.a	.a	.a	.a	.a	.a	.a	.a	.a	.a	.a	.a	.a	.a	.a	.a	.a	.a	.a	.a	.a	.a
	Sig. (2-tail	.	.	.	.	.	.	.	.	.	.	.	.	.	.	.	.	.	.		.	.	.	.	.	.	.	.
	N	156	156	156	156	156	156	156	156	156	156	156	156	156	156	156	156	156	156	156	156	156	156	156	156	156	156	156
sneezing	Pearson C	-0.07	0.095	-0.043	-0.111	-0.03	0.047	-0.152	0.014	0.073	0.058	0.102	0.041	0.153	0.007	.162*	.337**	0.047	-0.086	.a	1	-0.05	-0.007	-0.05	-0.135	0.071	0.005	-0.01
	Sig. (2-tail	0.382	0.239	0.597	0.168	0.712	0.56	0.058	0.867	0.367	0.471	0.204	0.61	0.056	0.926	0.044	0	0.56	0.287	.		0.539	0.929	0.539	0.093	0.377	0.951	0.904
	N	156	156	156	156	156	156	156	156	156	156	156	156	156	156	156	156	156	156	156	156	156	156	156	156	156	156	156
kissing no	Pearson C	0.027	-0.049	0.066	0.072	0.037	0.029	0.06	-0.009	-0.057	0.084	0.052	-0.079	0.144	-.161*	0.088	-0.063	0.055	-0.094	.a	-0.05	1	0.065	-0.043	-0.11	-0.028	0.015	0.04
	Sig. (2-tail	0.74	0.543	0.415	0.372	0.647	0.72	0.459	0.91	0.477	0.295	0.516	0.33	0.073	0.044	0.276	0.438	0.493	0.244	.	0.539		0.42	0.596	0.171	0.728	0.857	0.624
	N	156	156	156	156	156	156	156	156	156	156	156	156	156	156	156	156	156	156	156	156	156	156	156	156	156	156	156
oral care	Pearson C	0.106	0.071	0.049	0.047	.168*	-0.055	0.071	.180*	0.001	0.119	-0.023	-0.034	0.119	0.076	.192*	0.13	0.135	0.085	.a	-0.007	0.065	1	-0.07	0.042	0.021	-0.071	-.202*
	Sig. (2-tail	0.187	0.377	0.542	0.557	0.036	0.495	0.382	0.025	0.993	0.14	0.778	0.671	0.138	0.348	0.016	0.106	0.093	0.294	.	0.929	0.42		0.387	0.604	0.798	0.381	0.011
	N	156	156	156	156	156	156	156	156	156	156	156	156	156	156	156	156	156	156	156	156	156	156	156	156	156	156	156
bronchosc	Pearson C	.162*	0.081	.194*	.254**	0.131	-0.05	0.06	0.018	0.024	0.084	-0.055	0.133	0.144	0.035	0.021	.172*	0.082	-0.016	.a	-0.05	-0.043	-0.07	1	.252**	-0.106	-0.037	-.160*
	Sig. (2-tail	0.044	0.317	0.015	0.001	0.103	0.536	0.459	0.821	0.761	0.295	0.494	0.099	0.073	0.662	0.792	0.031	0.312	0.843	.	0.539	0.596	0.387		0.001	0.187	0.645	0.046
	N	156	156	156	156	156	156	156	156	156	156	156	156	156	156	156	156	156	156	156	156	156	156	156	156	156	156	156
intubation	Pearson C	0.124	0.009	0.058	.184*	0.058	-0.062	-0.015	0.073	0.077	0.105	0.014	0.037	0.075	0.138	-0.008	-0.016	.173*	-0.048	.a	-0.135	-0.11	0.042	.252**	1	-0.071	-0.038	-0.01
	Sig. (2-tail	0.122	0.906	0.473	0.021	0.47	0.445	0.854	0.368	0.336	0.19	0.865	0.647	0.349	0.086	0.921	0.842	0.03	0.556	.	0.093	0.171	0.604	0.001		0.381	0.639	0.903
	N	156	156	156	156	156	156	156	156	156	156	156	156	156	156	156	156	156	156	156	156	156	156	156	156	156	156	156
dressing n	Pearson C	0.021	0.009	0.027	-0.006	0.061	0.018	0.001	0.055	0.015	-0.033	-0.014	-0.015	-0.004	-0.057	-0.006	-0.113	0.097	0.151	.a	0.071	-0.028	0.021	-0.106	-0.071	1	0.027	.202*
	Sig. (2-tail	0.798	0.908	0.738	0.938	0.447	0.822	0.989	0.497	0.851	0.684	0.865	0.85	0.965	0.482	0.943	0.159	0.229	0.059	.	0.377	0.728	0.798	0.187	0.381		0.735	0.011
	N	156	156	156	156	156	156	156	156	156	156	156	156	156	156	156	156	156	156	156	156	156	156	156	156	156	156	156
knowledg	Pearson C	-0.039	-0.087	-0.038	0.013	0.024	0.094	0.134	-0.1	-0.023	-0.116	0.005	-0.121	0.102	-0.026	0.089	0.08	0.015	-0.117	.a	0.005	0.015	-0.071	-0.037	-0.038	0.027	1	-0.051
	Sig. (2-tail	0.625	0.282	0.635	0.874	0.763	0.245	0.095	0.215	0.778	0.149	0.946	0.134	0.205	0.745	0.272	0.318	0.849	0.147	.	0.951	0.857	0.381	0.645	0.639	0.735		0.53
	N	156	156	156	156	156	156	156	156	156	156	156	156	156	156	156	156	156	156	156	156	156	156	156	156	156	156	156
how to dis	Pearson C	-.224**	-0.033	-0.113	0.015	-0.096	0.073	-0.036	-.210**	0.021	-0.019	-0.003	-0.061	-.168*	-0.12	-.205*	-.231**	-0.1	-0.063	.a	-0.01	0.04	-.202*	-.160*	-0.01	.202*	-0.051	1
	Sig. (2-tail	0.005	0.679	0.159	0.857	0.232	0.365	0.654	0.008	0.792	0.818	0.97	0.448	0.036	0.135	0.01	0.004	0.216	0.432	.	0.904	0.624	0.011	0.046	0.903	0.011	0.53	
	N	156	156	156	156	156	156	156	156	156	156	156	156	156	156	156	156	156	156	156	156	156	156	156	156	156	156	156

**. Correlation is significant at the 0.01 level (2-tailed).

a. Cannot be computed because at least one of the variables is constant.

*. Correlation is significant at the 0.05 level (2-tailed).

		Correlations			
			wears a mask while caring for patient suspected of TB	Type of mask recoded	how often do you wear mask practice recoded
Spearman's rho	wears a mask while caring for patient suspected of TB	Correlation Coefficient	.	.	.
		Sig. (2-tailed)	.	.	.

		N	156	156	156
	Type of mask recoded	Correlation Coefficient	.	1	0.059
		Sig. (2-tailed)	.	.	0.468
		N	156	156	156
	how often do you wear mask practice recoded	Correlation Coefficient	.	0.059	1
		Sig. (2-tailed)	.	0.468	.
		N	156	156	156
